# Supplementary material for: The Controversial Issue of Hypervitaminosis B12 as Prognostic Factor of Mortality: Global Lessons from a Systematic Review and Meta-Analysis
Source: Nutrients. 2025 Jun 30;17(13):2184. doi: 10.3390/nu17132184 (PMC12252038; doi:10.3390/nu17132184)
Supplement: Supplementary file 1 [file nutrients-17-02184-s001.zip › Suppl. Table S1. Assessment of risk of bias (final_June2025).pdf]

Supplemental Table S1. Assessment of risk of bias (ROBINS-E)

	Author (publication year)	Risk of bias due to **confounding**	Risk of bias arising from **measurement of the exposure**	Risk of bias in **selection of participants** into the study or into the analysis	Risk of bias due to **post-exposure interventions**.	Risk of bias due to **missing data**	Risk of bias arising from **measurement of the outcome**	Risk of bias in **selection of the reported results**
1	Argan [18]	4	4	3	5	3	4	2
2	Callaghan [19]	2	3	3	5	3	3	2
3	Chen [20]	3	3	3	5	3	3	2
4	Couderc [3]	2	3	3	5	2	3	2
5	Dangour [21]	2	3	3	5	4	3	2
6	Dou [4]	2	3	3	5	5	3	2
7	Duschek [5]	3	3	3	5	3	3	2
8	Eduin [6]	3	3	3	1	3	4	1
9	Flores-Guerero [7]	3	2	3	5	3	3	2
10	Gamarra-Morales [22]	1	2	2	1	1	2	1
11	Geissbühler [8]	3	2	3	5	2	4	1
12	González [23]	3	3	3	1	3	4	2
13	Gonipath [24]	3	3	4	5	3	4	3
14	Huang [25]	2	3	2	5	3	2	2
15	Jia [26]	2	3	3	5	1	3	2
16	Lacombe [9]	2	4	4	5	3	4	3
17	Liu Y [10]	2	3	2	5	4	3	3
18	Mendoca [11]	3	3	3	5	3	3	2
19	Pusceddu [27]	2	3	2	5	2	3	4
20	Sviri [12]	2	3	3	1	5	3	1
21	Tal [13]	2	3	3	5	2	3	1
22	Valdivia [14]	2	3	3	5	3	4	1
23	Wang [28]	2	3	2	5	3	3	3
24	Wolffenbuttel [15]	1	3	2	5	2	3	1
25	Wu S [29]	3	3	3	5	3	3	2
26	Zhang [30]	2	3	3	4	5	3	1
27	Zeitlin [16]	3	3	4	5	4	4	3
28	Zhu [17]	4	4	3	5	5	4	2

1 = low risk of bias; 2 = some concerns; 3 = high risk of bias; 4 = very high risk of bias; 5 = it cannot be determined.

Supplemental Table S1. Assessment of risk of bias (ROBINS-E)—studies excluded

	Author (publication year)	Risk of bias due to **confounding**	Risk of bias arising from **measurement of the exposure**	Risk of bias in **selection of participants** into the study or into the analysis	Risk of bias due to **post-exposure interventions**.	Risk of bias due to **missing data**	Risk of bias arising from **measurement of the outcome**	Risk of bias in **selection of the reported results**
1	García-Rodríguez[1] (2019)	4	2	4	5	2	4	4
2	García-Rodríguez[2] (2021)	4	4	4	5	4	4	4

1 = low risk of bias; 2 = some concerns; 3 = high risk of bias; 4 = very high risk of bias; 5 = it cannot be determined.

[1] García-Rodríguez AM, Sánchez-Velasco MJ, Fernández-García N, Garrote-Adrados Ja. Hipervitaminosis B12: reactante y biomarcador en cáncer *(B12 hipervitaminosis: reactant and acute biomarker in cancer)*. Atención Primaria Práctica 2019;1(3):45-49. doi: 10.1016/j.appr.2019.01.002

[2] García-Rodríguez AM, Sánchez-Velasco MJ, González-Melgosa I, Villaescusa-Fulgencio C, Alonso-Díaz FA, Sánchez-Gómez E. Hipervitaminosis B12 y mortalidad a edades avanzadas. Atención Familiar 2021;28(1):26-32. doi: 10.22201/fm.14058871p.2021.1.77657
