# Supplementary material for: The Controversial Issue of Hypervitaminosis B12 as Prognostic Factor of Mortality: Global Lessons from a Systematic Review and Meta-Analysis
Source: Nutrients. 2025 Jun 30;17(13):2184. doi: 10.3390/nu17132184 (PMC12252038; doi:10.3390/nu17132184)
Supplement: Supplementary file 1 [file nutrients-17-02184-s001.zip › Suppl. Table S2. Summary table of characteristics of the studies included (june2025).pdf]

Supplemental Table S2. Summary table of characteristics of the studies included (n = 28).

Reference	Country of study	Study design	Study population and setting	Sample size at baseline and sampling	Attrition details	Exposure (B12 quantification) ascertainment	Vitamin B12 measurement technique and units, and cut-off points.	Follow-up	Outcome (mortality) ascertainment
Flores-Guerero (2020) [7]	Netherlands	A population-based retrolective cohort (of evolution of a group).	Noninstitutionalized Groningen civilian population. Average age 53.5 [SD 12.0] years.	n = 5,571, consecutive	Unreported	– One time only, at baseline. – Electronic database.	– Elecsys vitamin B12 II assay. – pg/mL – quarters	Median 8.2 [range: 7.7-8.9] years, from the baseline visit date.	Data linked to civil registry and vital statistics.
Geissbühler P (2000) [8]	Switzerland	A hospitalized-based prolective cohort (of evolution of a group).	Palliative care cancer patients from a geriatric facility. Median age 74.7 [range: 46-96] years.	n = 157, consecutive	Unreported	– One time only, at baseline. – Frozen serum samples.	– RIA technique. – pmol/L – thirds	5 years, from the enrolling date.	Data linked to civil registry and vital statistics.
Gonzalez S (2007) [23]	Spain	A population-based prolective cohort (of evolution of a group).	Institutionalized (Asturian nursing homes) civilian population. Average age 75.1 [SD 6.5] years.	n = 215, consecutive	n = 11, left alive.	– One time only, at baseline. – Blood samples analysed on the same day.	– Competitive -binding immunoenzymic assay. – pmol/L – fifths	Median 4.3 years, [range: no reported], from the date of the baseline visit.	Contacting the institutions (nursing homes).
Lacombe V (2021) [9]	France	A hospitalized-based retrolective cohort (of evolution of a group).	Patient records from Angers University Hospital. Median age 79 [range: 64-86] years.	n = 688, consecutive	Unreported *"Loss of follow-up was censored"*	Patients with ≥3 B12 measurements, that one ≥ 1000 was selected as the 1st test. In the absence of B12 ≥ 1000, the 1st test was randomly selected between the 1st and penultimate measurement.	– Immunoanalytical system ADVIA centaur – ng/L – two halves	Median 3.2 [range: 1.5-5.2] years, unclear the starting point of the following-up.	Patient records
Sviri S (2012) [12]	Israel	A hospitalized-based retrolective cohort (of evolution of a group).	Patient records from the Medical Intensive Care Unit (MICU) at the University Medical Centre in Jerusalem. Average age 60.4| [SD 20.4] years.	n = 663, consecutive	Unreported	– One time only, at baseline. – Electronic database.	– Chemiluminescent enzyme immunoassay. – pg/mL – two halves	Median 90 days, [range: no reported], from the admission date to the MICU.	Data linked to civil registry and vital statistics, and MICU database.
Valdivia G (2020) [14]	Chile	A hospitalized-based prolective cohort (of evolution of a group).	Patients admitted to the internal medicine service of the Hospital Victor Rios Ruiz in Los Angeles. Average age 80 [SD 3.6] years.	n = 93, consecutive	Unreported	– One time only, at baseline. – Blood samples analysed on the same day.	– Unreported technique. – pg/mL – two halves	12 months, from the hospital admission date.	Data linked to civil registry and vital statistics, and clinical records.
Wolffenbuttel B (2020) [15]	US	A population-based retrolective cohort (of evolution of a group) linked national health survey study.	Noninstitutionalized US civilian population. Average age 48 [SD 19] years.	n = 24,262 consecutive	Unreported	– One time only, at baseline. – Electronic database.	– Quantaphase II radioimmunoassay for the 1999-2006 surveys. – Electrochemiluminescence immunoassay for the 2011-2014 surveys. – pmol/L – quarters	Median 109 [range: 1-201] months, from the enrolling date.	Data linked to National Death Index.

Supplemental Table S2. Summary table of characteristics of the studies included *(Continued)*.

Reference	Country of study	Study design	Study population and setting	Sample size at baseline and sampling	Attrition details	Exposure (B12 quantification) ascertainment	Vitamin B12 measurement technique and units, and cut-off points.	Follow-up	Outcome (mortality) ascertainment
Callaghan F (2014) [19]	US	A hospitalized-based retrolective cohort (of evolution of a group).	Patient records from the Intensive Care Unit (ICU) at Boston's Beth Israel Deaconess Medical Centre. Median age 67.7 [range: 55.4-78.1] years.	n = 1,684, consecutive	Unreported	– One time only, at baseline.<br>– Electronic medical records.	– Unreported technique.<br>– pmol/L<br>– two halves	Median 90 days, [range: unreported], from the admission date to the ICU.	Electronic medical records.
Argan O (2018) [18]	Turkey	An outpatient-based prolective cohort (of evolution of two groups).	Heart failure (HF) patients admitted to the cardiology department of Kocaeli University. "Exposed group" (HF & high B12), Mean age 65 [SD 12] years. "Unexposed (healthy) group", Average age 49 [SD 12] years.	n = 179, consecutive<br>"Exposed" n=129<br>"Unexposed" n=50	Unreported	– One time only, at baseline.<br>– Blood samples analysed on the same day.	– Chemiluminescent enzyme immunoassay.<br>– pg/mL<br>– two halves	Median 32, [range: 17-44] months, from the admission date to the cardiology department.	By outpatient visits or by phone contact.
Liu Y (2022) [10]	US	A population-based retrolective cohort (of evolution of a group) linked national health survey study.	Noninstitutionalized US civilian population with type 2 diabetes. Average age 57.8 [SD 0.3] years.	n = 4,860, consecutive	Unreported	– One time only, at baseline.<br>– Electronic database.	– Quantaphase II radioimmunoassay for the 1999-2006 surveys.<br>– Electrochemiluminescence immunoassay for the 2011-2014 surveys.<br>– pg/mL<br>– quarters	43,855 person-years, from the enrolling date.	Data linked to National Death Index.
Wang S (2022) [28]	US	A population-based retrolective cohort (of evolution of a group) linked national health survey study.	Noninstitutionalized US civilian population with type 2 diabetes. Average age 59.3 [SD 0.3] years.	n = 3,277, consecutive	Unreported	– One time only, at baseline.<br>– Electronic database.	– Quantaphase II radioimmunoassay for the 1999-2004 surveys.<br>– Electrochemiluminescence immunoassay for the 2011-2014 surveys.<br>– pg/mL<br>– thirds	Median 7.02 years, [range: unreported], from the enrolling date.	Data linked to National Death Index.
Dou J (2012) [4]	China	A hospitalized-based prolective cohort (of evolution of a group).	Patients with acute-on-chronic liver failure admitted to the University Hospital in Zhejiang. Average age 47.2 [SD 11.2] years.	n = 105, consecutive	Unreported	– One time only, at baseline.<br>– Blood samples analysed on the same day.	– Chemiluminescent method.<br>– pg/mL<br>– thirds	Median 81, [range: 24-121] days, from the admission date to the hospital.	Medical records.
Eduin B (2023) [6]	France	A hospitalized-based retrolective cohort (of evolution two groups).	Patients records from the internal medicine department of University Hospital in Montpellier. Average age 77 [SD 15] years.	n = 330:<br>– high B12 n = 165, consecutive.<br>– normal B12 n = 165, randomly selected.	Unreported	– One time only, at baseline.<br>– Medical records of admission.	– Electrochemiluminescence assay.<br>– pmol/L<br>– two halves	12 months, from the admission date to the hospital.	Based on data from the national database.
Zhang P (2023) [30]	US	A population-based retrolective cohort (of evolution of a group) linked national health survey study.	Noninstitutionalized US civilian population with stroke. Average age 64.8 [SD 1.07] years.	n = 431, consecutive	Unreported	– One time only, at baseline.<br>– Electronic database.	– Quantaphase II radioimmunoassay for the 1999-2006 surveys.<br>– pmol/L<br>– quarters	Median 10.4 years, [range: unreported], from the enrolling date.	Data linked to National Death Index.

Supplemental Table S2. Summary table of characteristics of the studies included *(Continued)*.

Reference	Country of study	Study design	Study population and setting	Sample size at baseline and sampling	Attrition details	Exposure (B12 quantification) ascertainment	Vitamin B12 measurement technique and units, and cut-off points.	Follow-up	Outcome (mortality) ascertainment
Duschek N (2016) [5]	Austria	A hospitalized-based prolective cohort (of evolution of a group).	Patients undergoing carotid surgery at a vascular & endovascular surgery department in Vienna. Median age 71 [range: 63-78] years.	n = 485, consecutive	Unreported	– One time only, at baseline. – Blood samples analysed on the same day.	– Abbott AxSYM Plus immunology analyser. – pmol/L – fifths	102.3 months, from the surgery date.	Hospital registries cross-checked with the National public death registry.
Pusceddu I (2019) [27]	Germany	A hospitalized-based prolective cohort (of evolution of a group).	Patients for elective diagnostic coronary angiography at the Heart Centre Ludwigshafen. Median age 63.5 [range: 48.1-75.6] years.	n = 3,312, consecutive	Unreported	– One time only, at baseline. – Stored blood samples.	– Abbott AxSYM Plus immunology analyser. – pmol/L – quarters	Median 9.9 years, [range: unreported], from the enrolling date.	Local person registries.
Couderc AL (2020) [3]	France	An outpatient-based prolective cohort (of evolution of a group).	Patients with cancer at Marseille University Hospital. Median age 81 [range: 70-98] years.	n = 621, consecutive	n =51, left alive.	– One time only, at baseline. – Blood samples analysed on the same day.	– Unreported technique. – pmol/L – two halves	Median 10, [range: 1-35] months, from the diagnosis of the disease.	Medical records.
Jia X (2007) [26]	UK	A population-based prolective cohort (of evolution of a group).	Noninstitutionalized Aberdeen, Scotland, civilian population. Median age 80 [range: 75-96] years.	n = 398 random	Unreported	– One time only, at baseline. – Stored blood samples.	– Technicon Immuno 1 radioimmunoassay. – pmol/L – fifths	Median 69, [range: 1-79.9] months, from the enrolling date.	Grampian CHI cross-checked with the General Register Office for Scotland.
Dangour A (2008) [21]	UK	A population-based prolective cohort (of evolution of a group) as a part of a MRC trial[1].	Noninstitutionalized UK civilian population. Median age 78.6 [range: 76.8-81.2] years.	n = 853 random	Unreported	– One time only, at baseline. – Stored blood samples.	– SimulTRAC Assay, Becton Dickinson. – pmol/L – thirds	Median 7.6, [range: 4.5-8.5] years, from the enrolling date.	The Office for National Statistics.
Mendonça N (2018) [11]	UK	A population-based prolective cohort (of evolution of a group).	Noninstitutionalized Northeast England civilian population, aged 85+ years.	n = 749, consecutive	Unreported	– One time only, at baseline. – Analysis date of blood samples is unreported.	– Chemiluminescence immunoassay – pmol/L – quarters	9 years, from the blood draw.	Health & Social Care Information Service (now NHS digital).
Chen S (2021) [20]	Japan	A population-based prolective cohort (of evolution of a group).	Noninstitutionalized Hisayama civilian population. Average age 60 [SD 12] years.	n = 3,050, consecutive	*"No participants were lost to follow-up"*	– One time only, at baseline. – Frozen serum samples.	– Chemiluminescent enzyme immunoassay. – pmol/L – thirds	10.2 years, from the enrolling date.	Hospital records cross-checked with death certificates.
Zeitlin A (1997) [16]	US	A population-based retrolective cohort (of evolution of a group).	Noninstitutionalized Bronx, New York, civilian population, aged 75-85 years.	n = 440, consecutive	Unreported	– During initial and annual follow-up evaluations. – Analysis date of blood samples is unreported.	– Radioimmunoassay kit – pg/mL – average estimated by population groups.	Average 6 years [SD no reported], from the enrolling date.	Hospital records cross-checked with death certificates.

[1] MRC trial = Medical Research Council trial of assessment and management of older people in the community

Supplemental Table S2. Summary table of characteristics of the studies included *(Continued)*.

Reference	Country of study	Study design	Study population and setting	Sample size at baseline and sampling	Attrition details	Exposure (B12 quantification) ascertainment	Vitamin B12 measurement technique and units, and cut-off points.	Follow-up	Outcome (mortality) ascertainment
Gamarra -Morales Y (2022) [22]	Spain	A hospitalized-based prolective cohort (of evolution of a group).	Patient from the Intensive Care Unit (ICU) of University Hospital in Granada. Median age 61.9 [SD 14.1] years.	n = 28, consecutive	Unreported	– Twice, on days 1 and 3 of their stay in the UCI<br>– Blood samples analysed on the same day.	– Electrochemiluminescence immunoassay.<br>– pg/mL<br>– average estimated by population groups.	28 days, from the admission date to the ICU.	Unreported.
Zhu X (2023) [17]	US	A population-based retrolective cohort (of evolution of a group) linked national health survey study.	Noninstitutionalized US civilian population with hypertension. Average age 58.1 [SD 17.5] years.	n = 9,934, consecutive	Unreported	– One time only, at baseline.<br>– Electronic database.	– Quantaphase II radioimmunoassay for the 1999-2006 surveys.<br>– Electrochemiluminescence immunoassay for the 2011-2014 surveys.<br>– pg/mL<br>– quarters	Average 11 years [SD no reported], from the enrolling date.	Data linked to National Death Index.
Wu S (2023) [29]	US	A population-based retrolective cohort (of evolution of a group) linked national health survey study.	Noninstitutionalized US civilian population with chronic kidney disease. Average age 58.1 [SD 17.5] years.	n = 2,589, consecutive	Unreported	– One time only, at baseline.<br>– Electronic database.	– Quantaphase II radioimmunoassay for the 1999-2004 surveys.<br>– Electrochemiluminescence immunoassay for the 2011-2014 surveys<br>– pmol/L<br>– quarters	Average 7.7 years [SD no reported], from the enrolling date.	Data linked to National Death Index.
Huang YC (2012) [25]	US	A population-based retrolective cohort (of evolution of a group) linked national health survey study.	Noninstitutionalized Taiwanese civilian population, aged ≥65 years.	n = 1,297, consecutive	Unreported	– One time only, at baseline.<br>– Electronic database.	– Unreported technique<br>– pmol/L<br>– two halves	Median 7.4, [range: 0.02-9.8] years, from the enrolling date.	Data linked to National Death Registration.
Tal S (2023) [13]	Israel	An outpatient-based prolective cohort (of evolution of a group).	Patients discharged from acute geriatric ward of University Hospital in Jerusalem. Average age 93.1 [SD 3.3] years.	n = 448, consecutive	Unreported	– One time only, the time of B12 test not specified.<br>– Electronic hospital health records.	– Unreported technique<br>– pmol/L<br>– two halves	1 year after discharge.	Electronic hospital health records.
Gopinath B (2015) [24]	Australia	A population-based prolective cohort (of evolution of a group).	Noninstitutionalized Sidney civilian population with common eye diseases. Average age 58.1 [SD 17.5] years.	n = 2,963, consecutive	Unreported	– One time only, at baseline.<br>– Blood samples analysed on the same day.	– Competitive-binding assay method.<br>– pmol/L<br>– two halves	10 years, from the enrolling date.	Data linked to National Death Index.
