# Supplementary figures and images for: The Controversial Issue of Hypervitaminosis B12 as Prognostic Factor of Mortality: Global Lessons from a Systematic Review and Meta-Analysis
Source: Nutrients. 2025 Jun 30;17(13):2184. doi: 10.3390/nu17132184 (PMC12252038; doi:10.3390/nu17132184)

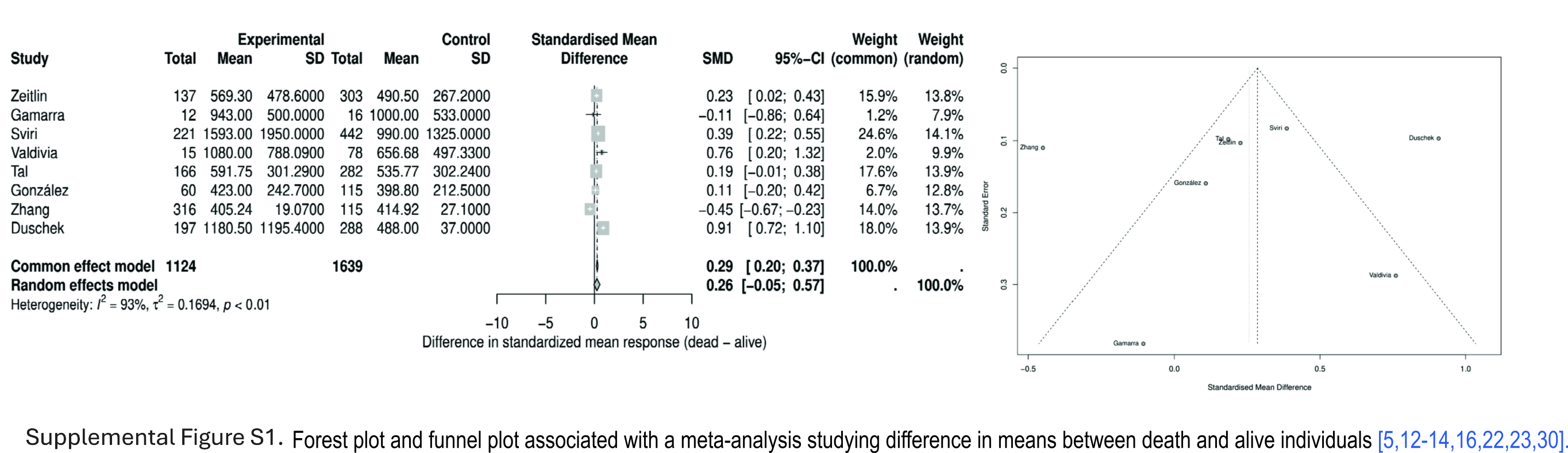

Supplement: Supplementary file 1 [file nutrients-17-02184-s001.zip › Suppl. Figure S1. Forest plot and funnel plot.jpg]

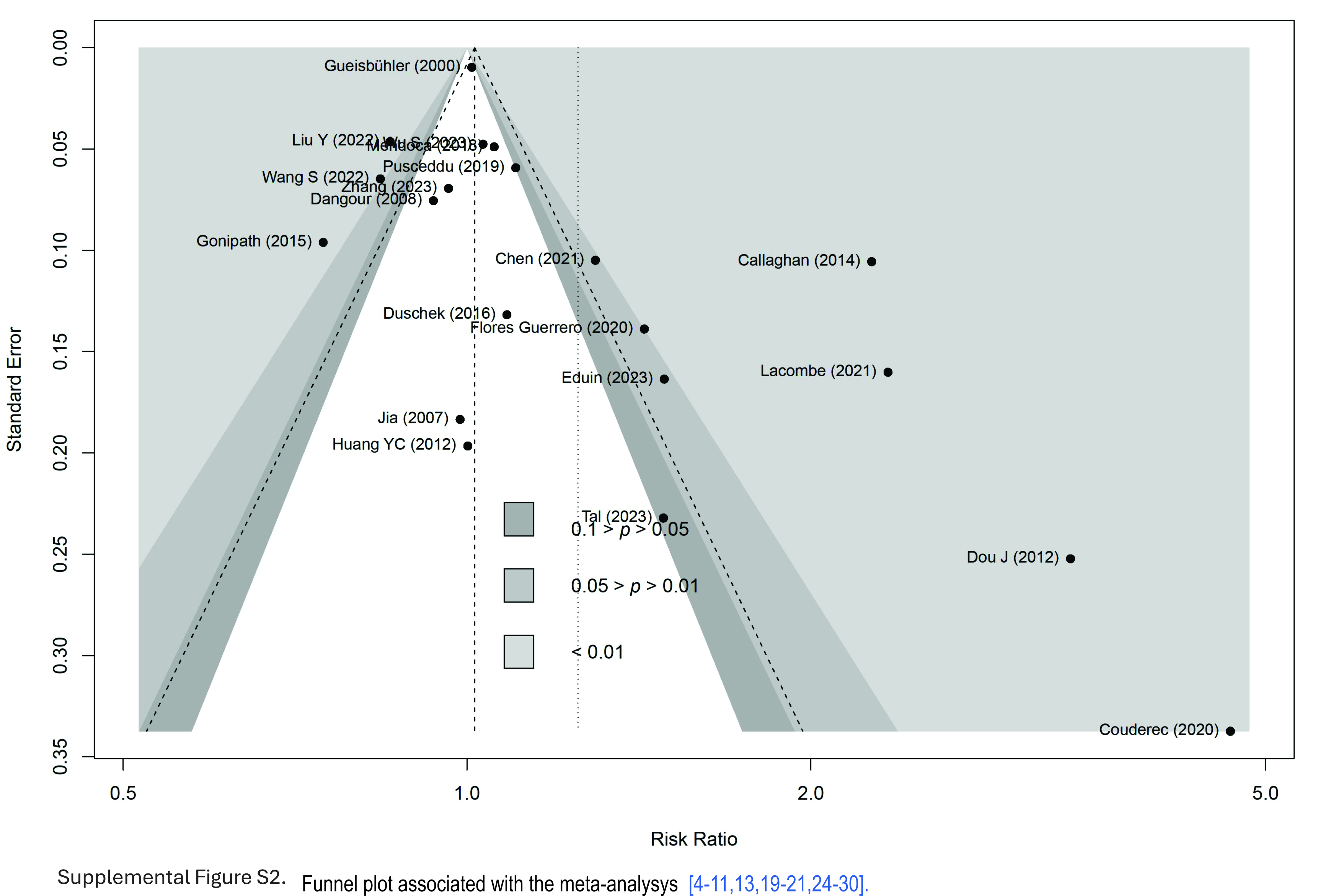

Supplement: Supplementary file 1 [file nutrients-17-02184-s001.zip › Suppl. Figure S2. Funnel plot.jpg]
